# Differential Mechanism of *Escherichia coli* Inactivation by (+)-Limonene as a Function of Cell Physiological State and Drug's Concentration

**DOI:** 10.1371/journal.pone.0094072

**Published:** 2014-04-04

**Authors:** Beatriz Chueca, Rafael Pagán, Diego García-Gonzalo

**Affiliations:** Tecnología de los Alimentos, Departamento de Producción Animal y Ciencia de los Alimentos, Facultad de Veterinaria, Universidad de Zaragoza, Zaragoza, Spain; University of Rochester, United States of America

## Abstract

(+)-limonene is a lipophilic antimicrobial compound, extracted from citrus fruits' essential oils, that is used as a flavouring agent and organic solvent by the food industry. A recent study has proposed a common and controversial mechanism of cell death for bactericidal antibiotics, in which hydroxyl radicals ultimately inactivated cells. Our objective was to determine whether the mechanism of *Escherichia coli* MG1655 inactivation by (+)-limonene follows that of bactericidal antibiotics. A treatment with 2,000 μL/L (+)-limonene inactivated 4 log_10_ cycles of exponentially growing *E. coli* cells in 3 hours. On one hand, an increase of cell survival in the Δ*acnB* mutant (deficient in a TCA cycle enzyme), or in the presence of 2,2′-dipyridyl (inhibitor of Fenton reaction by iron chelation), thiourea, or cysteamine (hydroxyl radical scavengers) was observed. Moreover, the Δ*recA* mutant (deficient in an enzyme involved in SOS response to DNA damage) was more sensitive to (+)-limonene. Thus, this indirect evidence indicates that the mechanism of exponentially growing *E. coli* cells inactivation by 2,000 μL/L (+)-limonene is due to the TCA cycle and Fenton-mediated hydroxyl radical formation that caused oxidative DNA damage, as observed for bactericidal drugs. However, several differences have been observed between the proposed mechanism for bactericidal drugs and for (+)-limonene. In this regard, our results demonstrated that *E. coli* inactivation was influenced by its physiological state and the drug's concentration: experiments with stationary-phase cells or 4,000 μL/L (+)-limonene uncovered a different mechanism of cell death, likely unrelated to hydroxyl radicals. Our research has also shown that drug's concentration is an important factor influencing the mechanism of bacterial inactivation by antibiotics, such as kanamycin. These results might help in improving and spreading the use of (+)-limonene as an antimicrobial compound, and in clarifying the controversy about the mechanism of inactivation by bactericidal antibiotics.

## Introduction

Although the antimicrobial properties of plant essential oils (EOs) have been recognized for thousands of years [1], their use in clinical, cosmetic, or food applications is a recent and growing trend reflecting the interest of producers and consumers to avoid synthetic drugs and preservatives. On the other hand, the occurrence of bacterial resistance to antibiotics [2] is stimulating the pharmaceutical industry to search for alternative antimicrobials.

(+)-limonene, the major chemical component of citrus fruits' EOs [3], [4], is widely used as a flavouring ingredient because of its citrus fruit flavor or organic solvent for industrial purposes [5], [6]. Apart from current applications, its use as a chemotherapeutic and chemopreventive compound [7], [8] or as a food preservative [9] due to its antimicrobial properties [10]–[16] has also been proposed. This compound belongs to the cyclic monoterpene hydrocarbon family, which is believed to accumulate in the microbial plasma membrane and, thus, cause the loss of membrane integrity and dissipation of the proton motive force [17]. The lethal action of (+)-limonene was considered under the “quantal” effect (“all or nothing”) [15], revealing a different mechanism of action between (+)-limonene and other EO compounds, such as citral or carvacrol [18], [19].

Interestingly, a relatively recent and revealing study by Kohanski et al. [20] demonstrated that all classes of bactericidal antibiotics share a common mechanism of cellular death, which is in contrast to the general belief that attributed the killing effect to the class-specific drug-target interactions. According to this mechanism, regardless of drug-target interaction, antibiotics trigger harmful hydroxyl radical formation by the activation of the tricarboxylic acid cycle (TCA) and the later conversion of NADH to NAD^+^ through the electron transport chain. Normal electron transport in *E. coli* is accompanied by the generation of reactive oxygen species (ROS), such as superoxide and hydrogen peroxide. In the next step of the mechanism proposed, ROS formed by respiration cause leaching of iron from iron-sulfur clusters and stimulation of the Fenton reaction. Hydroxyl radical is formed mainly through the Fenton reaction, in which ferrous iron transfers an electron to hydrogen peroxide [21], [22]. Finally, cell death occurs because hydroxyl radicals are extremely toxic and will readily damage proteins, membrane lipids, and DNA.

However, this common mechanism has been refuted by other authors [23], [24] who have concluded that ROS are not involved in cell death mediated by antibiotics, because modulation of their respective targets (inhibition of cell-wall assembly, protein synthesis, and DNA replication) is the actual cause of the the bactericidal antibiotics' lethality. To the best of our knowledge, involvement of oxidative stress in the mechanism of bacterial inactivation by essential oils has not been demonstrated.

With the increasing interest in EOs as antimicrobial compounds, a better understanding of the specific sequence of the events leading to cell death caused by EO constituents is needed for their application as antimicrobial compounds. In consideration of these premises, we decided to investigate whether the mechanism of inactivation by (+)-limonene also follows the mechanism described by Kohanski et al. [20], or whether this mechanism is not valid for lipophilic antimicrobial compounds.

The aims of this work were: (a) to study the production of hydroxyl radical following exposure to bactericidal concentrations of (+)-limonene; (b) to confirm the presence of DNA damage following exposure of (+)-limonene by disabling the DNA damage response system (SOS response); (c) to study the relation between the mechanism of microbial inactivation by (+)-limonene and the tricarboxylic acid (TCA) cycle, the Fenton reaction, and iron source; (d) to determine the role of (+)-limonene concentration on its mechanism of inactivation; and (e) to evaluate the resistance of stationary-phase cells to (+)-limonene.

## Materials and Methods

### Micro-organisms and growth conditions

The strains used *Escherichia coli* MG1655 (ATCC 700926) and its derived strains Δ*recA*, Δ*acnB*, Δ*icdA*, Δ*sucB*, Δ*mdh*, Δ*tonB*, and Δ*iscS*, were provided by Collins Lab from Boston University [20]. During this investigation, the cultures were maintained in cryovials at −80°C. Broth subcultures were prepared by inoculating, with one single colony from a plate, a test tube containing 5 mL of sterile Luria Bertani Broth (LB; Sigma-Aldrich Steinheim, Germany). After inoculation, the tubes were incubated overnight at 37°C and then diluted 1∶500 in 25 mL of LB broth in 250 mL Erlenmeyer flasks. Exponential-phase cells were prepared by incubating the 250 mL-flasks under agitation (130 rpm; Selecta, mod. Rotabit, Barcelona, Spain) at 37°C in the dark until an optical density (OD_595_) of approximately 0.3 was reached, as measured using the spectrophotometer (Biochrom, mod. Libra S12, Cambridge, England). Stationary-phase cultures were prepared by incubating these flasks for 24 h under agitation at 37°C in the dark.

### Bacterial treatment with (+)-limonene and kanamycin

(+)-limonene (97% purum) was purchased from Sigma-Aldrich. This compound is practically immiscible in water, so a vigorous shaking method by vortex agitation (Genius 3, Ika, Königswinter, Germany) was used to prepare suspensions [25]. A stock solution of 50 mg/mL of kanamycin (Sigma-Aldrich) in water was prepared before the experiments.

For the exponential-phase experiments, (+)-limonene was added at final concentrations of 1,000, 2,000, and 4,000 μL/L and kanamycin was added at final concentrations of 3 and 5 μg/mL. These compounds were added to Erlenmeyer flasks containing 25 mL of LB with exponential-phase cultures, and they were maintained under agitation (130 rpm) at 37°C in the dark for 3 h.

For the stationary-phase experiments, the treatment medium was prepared by adding 2,000 μL/L (+)-limonene to tubes containing 10 mL of spent LB medium; this was the filter-sterilized supernatant obtained after centrifugation of a 24-h-grown culture. Before treatments, stationary-phase cultures were centrifuged at 6,000•*g* for 5 min and re-suspended in spent LB medium. Microorganisms were added at a final concentration of 10^8^ CFU/mL and maintained under constant agitation (130 rpm) at 25°C in the dark.

### Iron chelator and hydroxyl radical quenching experiments

2,2′-dipyridyl (Sigma-Aldrich) was added at a concentration of 500 μM. The application of iron chelators, such as 2,2′-dipyridyl, is an established means of blocking Fenton reaction-mediated hydroxyl radical formation by sequestering unbound iron [21].

Thiourea (Sigma-Aldrich) or cysteamine (Sigma-Aldrich) were added to achieve a final concentration of 150 mM and 2 μM, respectively. Thiourea is a potent hydroxyl radical scavenger and is often used to mitigate the effects of hydroxyl radical damage [26]–[29]. Cysteamine is a sulfhydryl compound and a hydroxyl radical scavenger [30], [31], which has also been found to be capable of chemical repair or modification of DNA damage [32].

Thiourea in solid form was weighed and added to the culture, whereas stock solutions of 500 μM of 2,2′-dipyridyl in ethanol (Merck, Darmstadt, Germany) and 2 M of cysteamine in Phosphate Buffered Saline, pH 7.3 (PBS; Oxoid, Hampshire, England), were previously prepared. 2,2′-dipyridyl, thiourea and cysteamine were added to the culture simultaneously with (+)-limonene.

The growth data in the presence of each hydroxyl radical scavenger alone was evaluated. Whereas cultures grown in the presence of 2 mM of cysteamine reached the same levels as control tubes (1 log_10_ cycle in 3 h), 150 mM of thiourea slowed down the bacterial growth rate in 0.5 log_10_ cycles (data not shown).

### Survival counts

Samples were taken every hour for 3 hours after the (+)-limonene addition: 100 μL of culture was collected and washed twice with filtered PBS. Samples were then serially diluted in PBS. 100 μL samples were pour-plated onto Luria Bertani Agar (LB agar; Sigma-Aldrich). Plates were incubated at 37°C in the dark for 24 h. Previous experiments showed that longer incubation times did not influence the survival counts.

After plate incubation, the colonies were counted with an improved image analyzer automatic counter (Protos; Analytical Measuring Systems, Cambridge, United Kingdom), as it had been previously described [33].

### Statistical analysis

Inactivation was expressed in terms of the extent of the reduction in log_10_ counts after every treatment. The error bars in the figures indicate the mean ± standard deviations from the data obtained from at least three independent experiments. ANOVA and t-tests were performed with GraphPad PRISM (GraphPad Software, Inc., San Diego, USA) and differences were considered significant if *p*≤0.05.

## Results

### Involvement of hydroxyl radicals in *E. coli* inactivation by (+)-limonene

**Figure 1:**
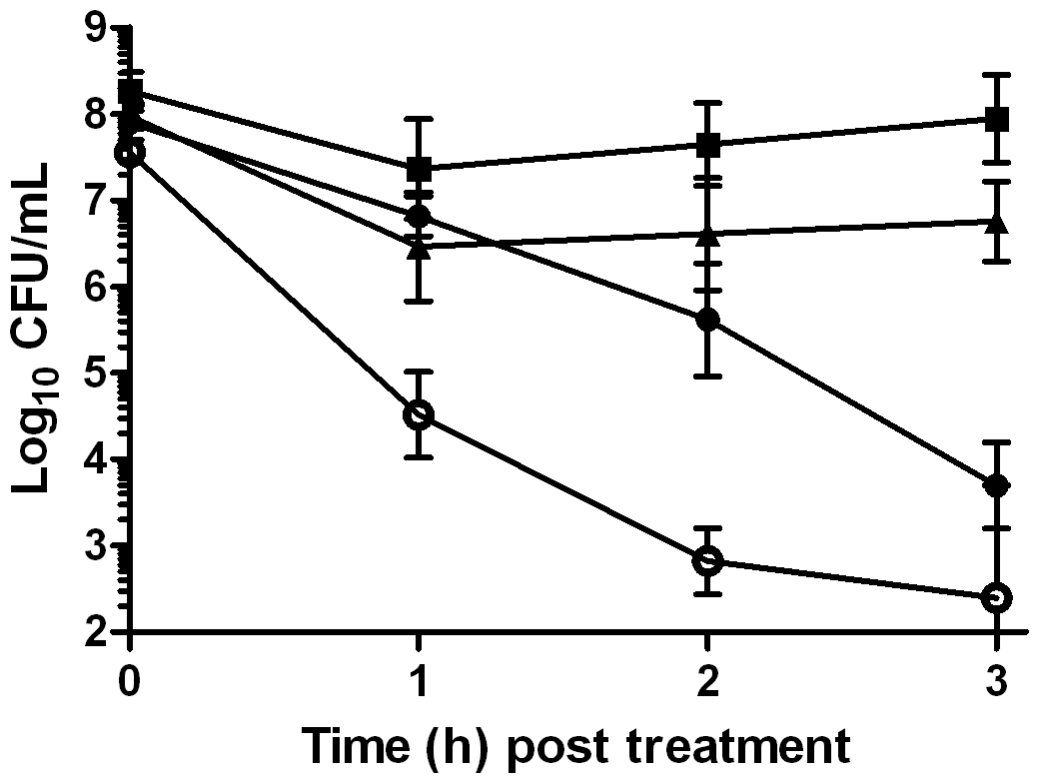
Involvement of hydroxyl radicals in *Escherichia coli* inactivation by (+)-limonene. Log_10_ of survival counts of exponential-phase cells of *Escherichia coli* MG1655 (closed symbols) and *E. coli* MG1655 Δ*recA* (○) treated with 2,000 μL/L of (+)-limonene in LB broth at 37°C (• and ○) and following the addition of 150 mM thiourea (▴) or 2 mM cysteamine (▪) in the wild-type cells. Cells were recovered in LB agar. Data are means ± standard deviations (error bars).

Figure 1 shows the (+)-limonene (2,000 μL/L) inactivation of exponential-phase *E. coli* MG1655 cells. For example, after 3 hours, a 4 log_10_ reduction in the number of viable cells was observed.

To check whether the (+)-limonene eventually would lead to the formation of hydroxyl radicals, we added ROS scavengers to the treatment medium. An evaluation of the survivors after 3 h showed a reduction of *E. coli* inactivation in 3 or 4 log_10_ cycles when either thiourea or cysteamine (*p*<0.05), respectively, was added to the medium simultaneously with (+)-limonene (Figure 1).

In order to evaluate the role of SOS response in (+)-limonene bacterial survival, its efficacy was tested in a *recA* knockout. Figure 1 illustrates that a decreased (+)-limonene resistance in Δ*recA* mutant was observed (*p*<0.05). While after 2 hours of 2,000 μL/L (+)-limonene treatment had killed 2 log_10_ wild-type cells, more than 4 log_10_ of Δ*recA* cells had been killed (Figure 1).

### Role of TCA cycle and iron in the mechanism of inactivation of (+)-limonene

**Figure 2:**
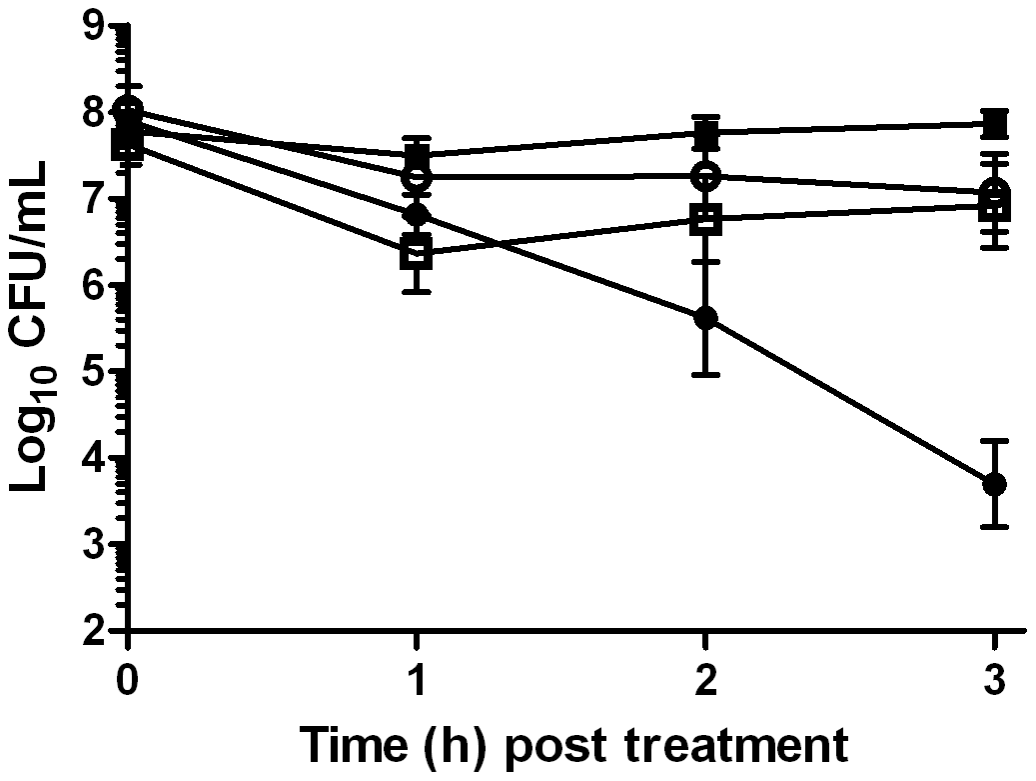
Role of TCA cycle and iron in the mechanism of *Escherichia coli* inactivation by (+)-limonene. Log_10_ of survival counts of exponential-phase cells of *Escherichia coli* MG1655 treated with 2,000 μL/L of (+)-limonene (•) following the addition of 500 μM 2,2′-dipyridyl (▪); *E. coli* MG1655 Δ*tonB* (□) and *E. coli* MG1655 Δ*acnB* (○)treated with 2,000 μL/L of (+)-limonene in LB broth at 37°C. Cells were recovered in LB agar. Data are means ± standard deviations (error bars).

The role of the TCA cycle in (+)-limonene-mediated cell death was evaluated with 4 knockout strains for TCA cycle component genes. Compared with the resistance of wild-type cells, Figure 2 shows an increase of cell survival in 3 log_10_ cycles by blocking the TCA cycle at the level of AcnB (*p*<0.05). The deletion of *icdA*, *sucB*, and *mdh* did not cause an important increase in survival (*p*>0.05; data not shown).

The addition of the iron chelator 2,2′-dipyridyl increased 4 log_10_ cycles the bacterial survivors to a 2,000 μL/L (+)-limonene treatment for 3 h (*p*<0.05; Figure 2). To determine whether the iron source was extracellular or intracellular, iron import was disabled by deleting the iron transporter gene *tonB*. Thus, figure 2 shows that the deletion of *tonB* increased cell resistance in 3 log_10_ cycles (*p*<0.05).

### Influence of (+)-limonene and kanamycin concentration in mechanism of bacterial inactivation

**Figure 3:**
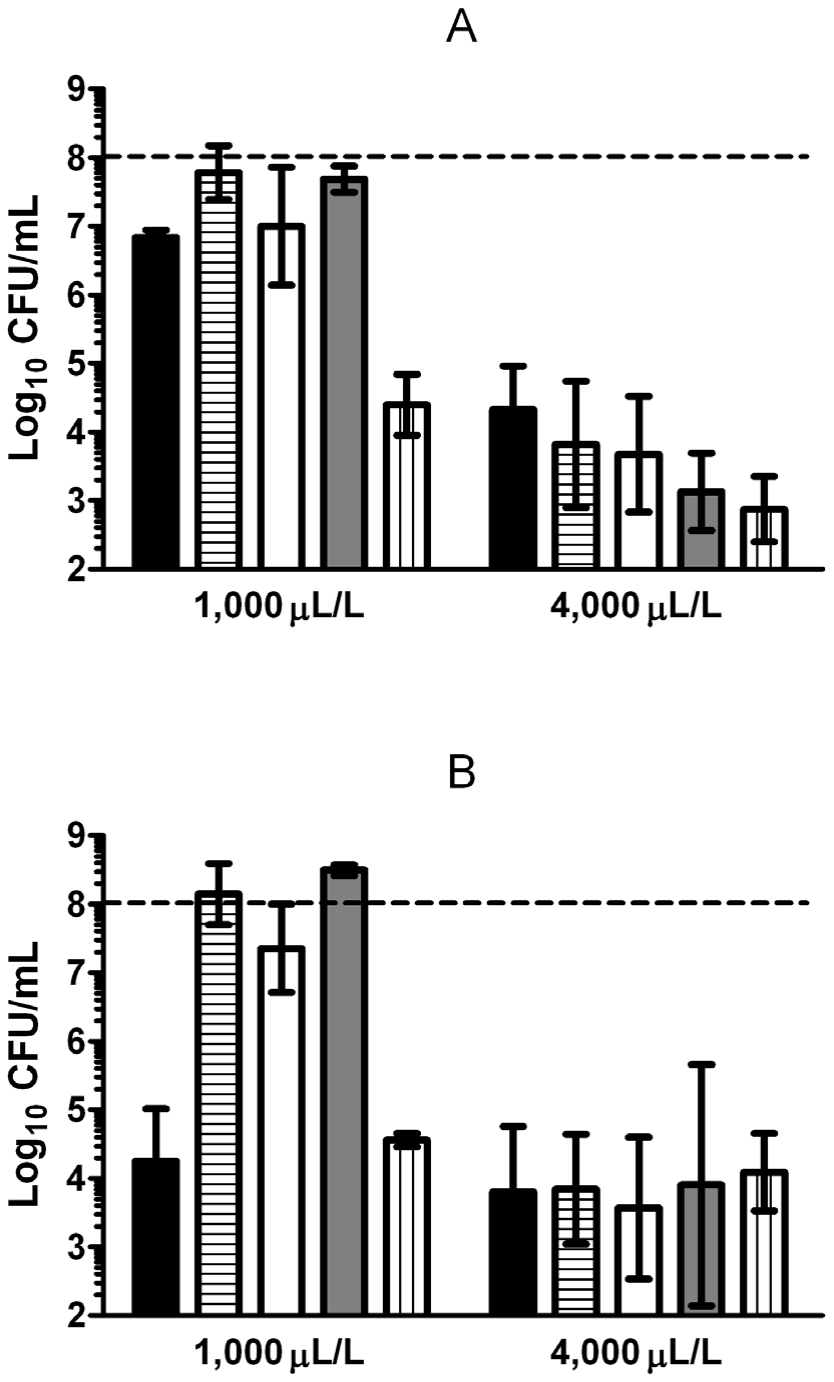
Influence of (+)-limonene concentration in the mechanism of bacterial inactivation. Log_10_ of survival counts after 1 (A) and 3 (B) hours of treatment with (+)-limonene in LB broth at 37°C of exponential-phase cells of *Escherichia coli* MG1655 (black bars) and following the addition of 500 μM 2,2′-dipyridyl (horizontal stripes), 150 mM thiourea (white bars), or 2 mM cysteamine (grey bars) and of *E. coli* MG1655 Δ*recA* (vertical stripes). Cells were recovered in LB agar. Discontinuous line indicates initial cell concentration (10^8^ CFU/mL). Data are means ± standard deviations (error bars).

The influence of the studied parameters on bacterial survival varied with the concentration of (+)-limonene (1,000, 2,000 and 4,000 μL/L). After 1 hour of treatment, only 1 log_10_ cycle of inactivation was reached with 1,000 and 2,000 μL/L of (+)-limonene, while 4 log_10_ cycles were achieved by treatment with 4,000 μL/L (Figure 3A). However, the inactivation level reached after 3 hours of treatment was similar with 1,000, 2,000, and 4,000 μL/L of (+)-limonene (*p*>0.05; Figure 3B), when 4 log_10_ cycles of inactivation were detected.

Whereas bacterial survival increased in the presence of thiourea, cysteamine, and 2,2′-dipyridyl for treatments with 1,000 and 2,000 μL/L of (+)-limonene, these compounds could not protect bacteria treated with 4,000 μL/L of (+)-limonene (*p*>0.05; Figure 3B). Bacterial resistance to 4,000 μL/L (+)-limonene in the presence of higher concentrations of thiourea (300 and 500 mM) was not modified (*p*>0.05; data not shown).

**Figure 4:**
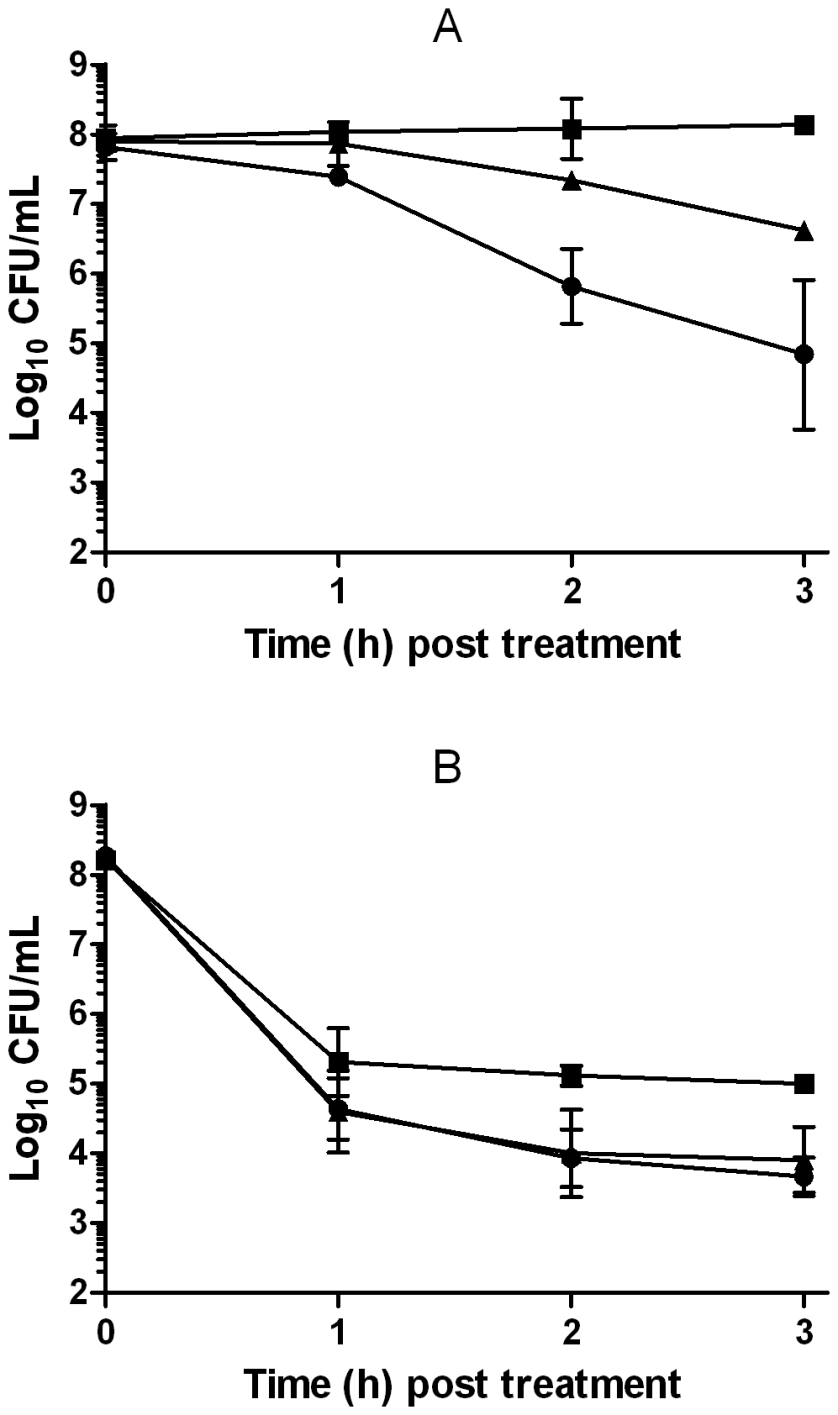
Resistance of *Escherichia coli* cells to kanamycin and influence of scavengers. Log_10_ of survival counts of exponential-phase cells of *Escherichia coli* MG1655 treated with 3 μg/mL (A) and 5 μg/mL (B) of kanamycin (•) and following the addition of 150 mM thiourea (▴) or 500 μM 2,2′-dipyridyl (▪). Cells were recovered in LB agar. Data are means ± standard deviations (error bars).

In order to compare these results with those observed in literature for bactericidal antibiotics, influence of kanamycin concentration on bacterial survival was also determined. As shown in Figure 4, after 3 hours of treatment with 3 and 5 μg/mL of kanamycin, more than 3 and 4 log_10_ cycles of *E. coli* inactivation were achieved, respectively. As observed for (+)-limonene, while at low drug concentrations thiourea and 2,2′-dipyridil increased cell survival in around 2 log_10_ cycles (Figure 4A); at higher drug concentrations, these scavenging agents decreased their ability to protect *E. coli* cells to kanamycin (Figure 4B).

### Stationary-phase cells experiments

**Figure 5:**
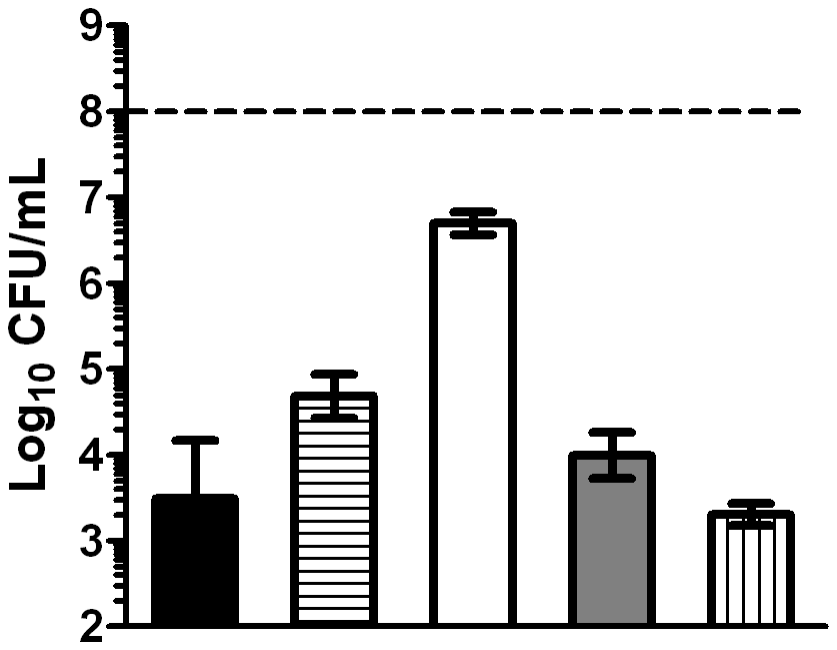
Resistance of stationary-phase *Escherichia coli* cells to (+)-limonene and influence of scavengers. Log_10_ of survival counts after 3 hours of treatment with 2,000 μL/L of (+)-limonene of stationary-phase cells of *Escherichia coli* MG1655 (black bars) and following the addition of 500 μM 2,2′-dipyridyl (horizontal stripes), 150 mM thiourea (white bars) or 2 mM cysteamine (grey bars) and of *E. coli* MG1655 Δ*recA* (vertical stripes). Cells were recovered in LB agar. Discontinuous line indicates initial cell concentration (10^8^ CFU/mL). Data are means ± standard deviations (error bars).

Survival of stationary-phase cells after 3 hours of treatment with 2,000 μL/L of (+)-limonene, shown in Figure 5, indicated a similar resistance to (+)-limonene between stationary- and exponential-phase cells (*p*>0.05). In contrast to exponentially growing cells, 2,2′-dipyridyl and cysteamine failed to protect cell death of wild-type cells at the stationary phase (*p*>0.05). However, thiourea increased bacterial survival in 3 log_10_ cycles, as observed in the exponential-phase cells (*p*<0.05). A *recA* mutant showed a similar level of inactivation as wild-type cells (*p*>0.05). These results suggest that the mechanism of inactivation for stationary-phase cells did not follow that observed for exponential-phase cells.

## Discussion

This study has demonstrated for the first time the lethal activity of (+)-limonene in actively growing *E. coli* MG1655 cells (Figure 1), as previously observed in stationary-phase cells in buffer [15]. Moreover, following the experimental protocol described by Kohanski et al. [20], the protection observed by thiourea to (+)-limonene-mediated cell death suggested a role of hydroxyl radical formation in the mechanism of the inactivation of exponentially growing cells. Nevertheless, thiourea slowed down *E. coli* growth under our treatment conditions and, as pointed out by some authors [23], [24], this reduction on cell metabolism might lead to increased tolerance toward antimicrobials such as antibiotics. Since thiourea scavenging activity and measurement of hydroxyl radical formation by fluorescein based dyes had been questioned [23], [24], we decided to use a different ROS scavenger, such as cysteamine, that did not modify the bacterial growth rate at the concentrations used in this study (data not shown). The use of cysteamine allowed us to confirm that the protection achieved could not be attributed to a reduction of growth and/or metabolic rates but, rather, to its role as ROS scavenger, as shown by Kohanski et al. [20]. As a consequence, this indirect evidence show that treatment with 2,000 μL/L (+)-limonene would lead to the formation of hydroxyl radicals in *E. coli* cells. Nevertheless, although both ROS scavengers demonstrated a different behaviour on cell growth rate, it cannot be discarded that cell protection caused by these compounds could impair (+)-limonene action by other indirect mechanisms, apart from radical scavenging. It is the first time that an essential oil compound has been suggested to cause formation of ROS leading to bacterial death in exponentially growing cells, as previously described for yeasts [34].

ROS have been shown to cause damage to DNA, RNA, proteins, and lipids [35]. This type of DNA damage is also called “oxidative damage to DNA”, and it results in lethal double-strand breaks and mutations [36]. *E. coli* has a number of complex enzymatic pathways for the repair of sublethal damages. For example, RecA serves as a regulatory protein to induce the SOS response to DNA damage, and it is a required component for the mutagenic bypass of DNA lesions during the SOS response [37], [38]. A decreased (+)-limonene resistance of a *recA* knockout would indicate DNA damage caused by this antimicrobial compound. Actually, Kohanski et al. [20] showed a correlation between the activation of SOS response (DNA damage) and the resistance of *recA* mutant to bactericidal antibiotics. Although *recA* deletion could influence other metabolic pathways leading to a decreased microbial resistance, the increased cell death showed by the Δ*recA* strain highlights the importance of an intact DNA damage repair system for mitigating the effects of hydroxyl radical-mediated DNA damage induced by (+)-limonene.

According to Kohanski et al. [20], NADH production via the TCA cycle is involved in antibiotic-mediated cell death, as it stimulates the increase of ROS (superoxide and hydrogen peroxide) via the electron transport chain. An increased resistance to (+)-limonene demonstrated in the Δ*acnB* strain, one of the knockout strains for the TCA cycle component genes showed the relevance of a normal activity of the TCA cycle in the (+)-limonene mechanism of action. AcnB catalyzes the reversible isomerization of citrate and isocitrate via cis-aconitate in the citric acid cycle and, among the four studied enzymes, it is located in the first step in TCA cycle. In contrast, resistance of Δ*icdA* and Δ*sucB* cells was similar to that observed in wild-type cells. This similar resistance could be explained by the existence of an alternative route, the glyoxylate cycle, which leads to NADH synthesis without the involvement of IcdA or SucB [39].

Therefore, as observed for bactericidal drugs by Kohanski et al. [20], the mechanism of inactivation by (+)-limonene seemed to be mediated by the TCA cycle that would eventually promote hydroxyl radical formation, leading to oxidative DNA damage, as observed in bactericidal drugs.

The production of hydroxyl radical occurs by the Fenton reaction, in which ferrous iron transfers electrons to hydrogen peroxide [26]. Therefore, hydroxyl radical stress increases when either hydrogen peroxide or ferrous concentrations are high [23]. The addition of the iron chelator 2,2′-dipyridyl increased bacterial survival (Figure 2), suggesting that iron is involved in bacterial (+)-limonene-induced cell death. The indirect evidence observed with 2,2′-dipyridyl and the hydroxyl radical scavengers would indicate that hydroxyl radical formation and the Fenton reaction both play a critical role in effective killing by (+)-limonene, as observed in bactericidal antibiotics [20].

**Figure 6:**
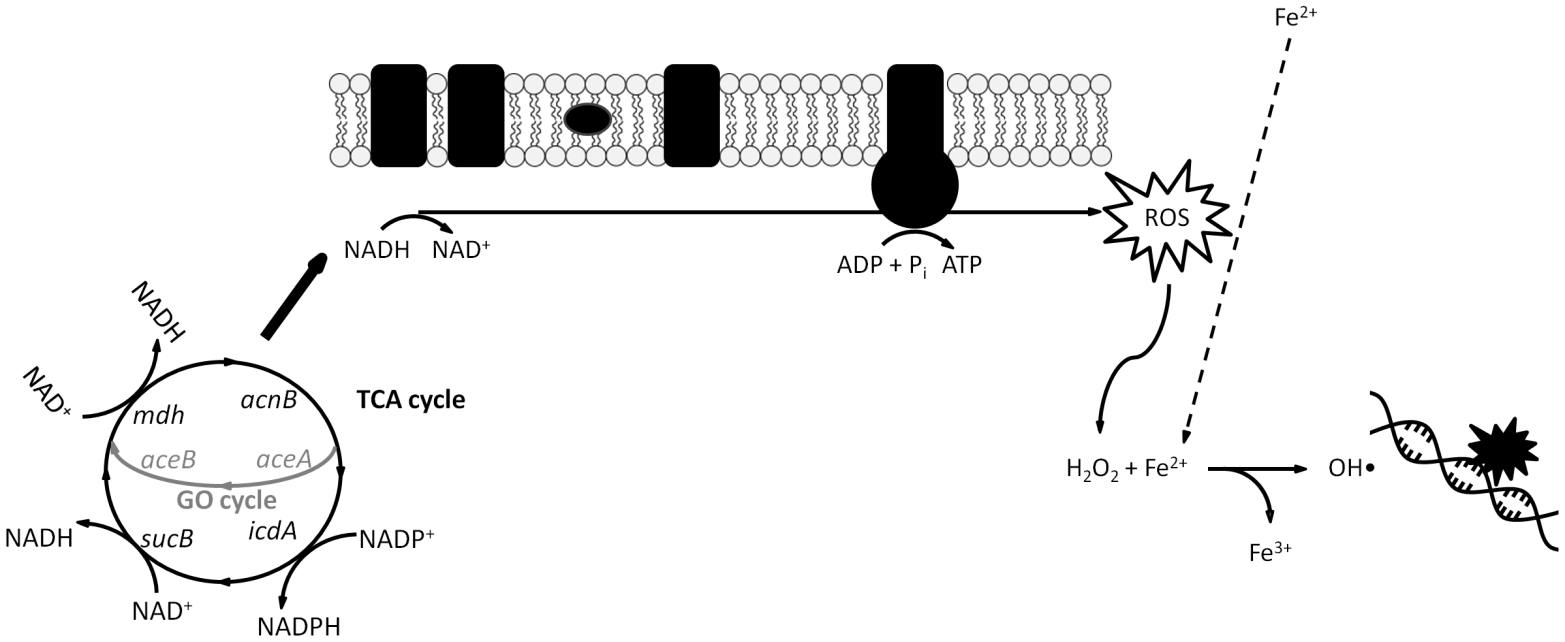
Proposed model for the mechanism of inactivation of *Escherichia coli* MG1655 exponential-phase cells by 2,000 μL/L (+)-limonene. (Based on Kohanski et al. [20]). 2,000 μL/L (+)-limonene stimulates the depletion of NADH via the electron transport chain that is dependent upon the TCA cycle in exponentially growing cells of *Escherichia coli* MG1655. Hyperactivation of the electron transport chain stimulates ROS formation, including hydrogen peroxide. The Fenton reaction, which involves hydrogen peroxide and free iron, leads to hydroxyl radical formation, which damages DNA, proteins, and lipids, resulting in microbial inactivation. (TCA cycle: tricarboxylic acid cycle. GO cycle: glyoxylate cycle).

The ferrous ion required for hydroxyl radical formation could come from extracellular sources, such as iron import, or from intracellular sources, such as iron storage proteins or iron-sulfur clusters. To investigate the source of the iron required for the Fenton reaction, (+)-limonene resistance of a *tonB* knockout mutant was tested. TonB is a cytoplasmic membrane protein that provides the energy source required for the import of iron-siderophore complexes and vitamin B12 across the outer membrane [40], [41]. A higher (+)-limonene resistance of Δ*tonB* strain (Figure 2) demonstrated the relevance of the external iron import to the Fenton reaction performance and the production of the hydroxyl radical in effective killing by (+)-limonene, as observed by Touati et al. [27] in death by oxidant stress exogenously induced via the application of hydrogen peroxide. The behavior of the Δ*tonB* strain to (+)-limonene treatment differed from that observed for bactericidal antibiotics [20], in which the source of iron was intracellular as an *iscS* knockout, with impaired iron-sulfur-cluster synthesis capabilities, was more resistant to bactericidal antibiotics. Thus, Kohanski et al. [20] concluded that superoxide formed through the electron transport chain was damaging iron-sulfur clusters, releasing ferrous iron for a Fenton reaction. The Δ*iscS* mutant not only showed no protection to (+)-limonene, but it was also much more sensitive than the wild-type strain (data not shown). According to our results, (+)-limonene would trigger the mechanism of action by activating the TCA cycle, NADH depletion through the electron transport chain, superoxide and hydrogen peroxide production, and Fenton reaction involving iron from extracellular sources (Figure 6).

On the one hand, our research has also demonstrated that concentration of the antimicrobial compound determined its mechanism of action. Cell death and protection by the iron chelator and hydroxyl radical scavengers varied with the concentration of (+)-limonene (1,000, 2,000 and 4,000 μL/L) (Figure 3); therefore, this proves that the ROS-mediated mechanism of inactivation was a function of (+)-limonene concentration. The different behavior of (+)-limonene at higher concentrations, such as 4,000 μL/L, where iron chelator and hydroxyl radical scavengers were not effective (Figure 3), would indicate that this compound could be acting through more than one mechanism of inactivation that could mask ROS production at certain levels. Fluorescence studies with propidium iodide showed that cell envelopes of dead exponential-phase cells were permeabilized after 4,000 μL/L (+)-limonene treatments (data not shown). These results would suggest that, at high (+)-limonene concentrations, envelopes permeabilization of exponentially growing cells would be related to cell inactivation, as previously determined for stationary-phase *E. coli* cells [15].

This unexpected mechanism led us to consider whether the experimental conditions in previous studies could explain the previously described controversy on the ROS-mediated mechanism by bactericidal drugs [20], [23], [24]. Thus, it was decided to investigate the influence of drug concentration in the mechanism of action of a bactericidal antibiotic such as kanamycin, particularly whether this mechanism would vary at higher concentrations, as observed for (+)-limonene. Effectively, at low kanamycin concentrations (3 μg/mL), thiourea and 2,2′-dipyridil increased cell survival in around 2 log_10_ cycles, whereas at higher concentrations (5 μg/mL), these scavenging agents failed in protecting cells (Figure 4). This result confirms that, in effect, this situation could be the source of discrepancies among studies on the mechanism of inactivation by bactericidal antibiotics [23], [24]. Keren et al. [24] also pointed out the importance of the concentration of the bactericidal antibiotic used in relation to the effect of thiourea and conclusions stemming from data obtained with fairly low concentrations of antibiotics [20]. They examined the effect of thiourea on killing at a range of antibiotic concentrations that included clinically achievable levels, and they found that the protection at higher relevant levels disappeared, discarding hydroxyl radical as the cause of cell death. However, both theories are consistent: our research has shown that the kinetics of inactivation are key in the ROS-involved mechanism of death.

On the other hand, Kohanski et al. [20] demonstrated the common mechanism for cells in exponential growth phase, as it is usually the growth phase at which antimicrobials are more effective against bacterial cells for clinical applications. Nevertheless, we considered it was likewise interesting to corroborate this mechanism of inactivation for stationary-phase cells, as usually it is a cell physiological state of increased resistance to most stressing agents. Although the resistance of stationary-phase cells in the spent LB medium was similar to that of exponential-phase cells, neither the iron chelator nor the hydroxyl radical scavenger cysteamine protected stationary-phase cells from (+)-limonene action (Figure 5). Although thiourea reduced cell death in 3 log_10_ cycles, this protection could be due to further inhibition of cell metabolism by thiourea [23], [24], leading to an increased tolerance to killing. Consequently, death in the stationary growth phase would not be due to hydroxyl radical formation, because iron and the Fenton reaction would be unnecessary [42]. This observation could also explain the controversy on the role of ROS in microbial death by bactericidal antibiotics, since it could also be possible that the physiological state of *E. coli* cells might differ among different studies.

Our results show that the mechanism of inactivation by (+)-limonene would be mediated by ROS in exponentially growing cells, but not in cells at a stationary growth phase. Therefore, the cell physiological state could determine the predominance of one mechanism over the other. For instance, it has been suggested that the synthesis of RpoS-dependent transporters and membrane proteins in the stationary phase may play a role in counteracting the increased generation of ROS in aerobic respiration [43]. It could also imply, in agreement with the lack of protection by 2,2′-dipyridyl and cysteamine, that there is no oxidative damage in the stationary phase caused by 4,000 μL/L (+)-limonene. Furthermore, the resistance of stationary Δ*recA* and wild-type cells was similar. In this case, it should be noted that RecA repair of a DNA damage is unique to exponential-phase cells [42], and this could be the reason of this analogous behavior between the two strains. Furthermore, previous results on the mechanism of inactivation by (+)-limonene [15] pointed out lipopolysaccharide (LPS) as the target of (+)-limonene at pH 7.0 in stationary-phase cells of *E. coli* BJ4 and showed a correlation a direct relationship between inactivated and permeabilized cells.

The use of (+)-limonene as an antimicrobial agent requires, among others, a detailed knowledge of its mechanism of inactivation and of the influence of environmental factors in its activity. In this study, we provide new clues to understand the mechanism of bacterial inactivation of this EO compound.

## Conclusions

In this study, we suggest hat (+)-limonene EO constituent follows a similar mechanism of killing as described by Kohanski et al. [20] for bactericidal drugs, but only under specific conditions of drug concentration and a certain cell physiological state. Thus, the mechanism of inactivation by (+)-limonene for exponentially growing cells and 2,000 μL/L (+)-limonene is likely due to the utilization of iron to promote Fenton-mediated hydroxyl radical formation that caused oxidative DNA damage (Figure 6), as observed in bactericidal drugs. However, extracellular iron import was a key source of the iron required to stimulate Fenton-mediated hydroxyl radical formation in (+)-limonene mediated killing, that being the main difference with the mechanism proposed for bactericidal drugs, in which the source of iron was intracellular, from iron-sulfur clusters.

The influence of drug concentration and/or cell physiological state in the mechanism of action of bactericidal drugs described in this research could be the cause of any discrepancies between those theories supporting ROS-mediated mechanism [20] and those declining it [23], [24]. A deeper knowledge in the role of other factors in these mechanisms of inactivation would allow us to better understand the killing effect of bactericidal drugs.

From a practical point of view, our results suggest that (+)-limonene could be potentiated by targeting bacterial systems that remediate hydroxyl radical damage, including proteins involved in triggering the DNA damage response. Interestingly, (+)-limonene was equally active against cells in both the stationary and exponential growth phases.
